# Does density-dependent diversification mirror ecological competitive exclusion?

**DOI:** 10.1371/journal.pone.0184814

**Published:** 2017-10-12

**Authors:** Melanie J. Monroe, Folmer Bokma

**Affiliations:** 1 Department of Ecology and Environmental Science and IceLab, Umeå University, Umeå, Sweden; 2 Department of Ecology and Genetics, Evolutionary Biology Center, Uppsala University, Norbyvägen 18D, Uppsala, Sweden; 3 Department of Biological Sciences and the IRMACS center for interdisciplinary research, Simon Fraser University, Burnaby, BC, Canada; Seconda Universita degli Studi di Napoli, ITALY

## Abstract

Density-dependence is a term used in ecology to describe processes such as birth and death rates that are regulated by the number of individuals in a population. Evolutionary biologists have borrowed the term to describe decreasing rates of species accumulation, suggesting that speciation and extinction rates depend on the total number of species in a clade. If this analogy with ecological density-dependence holds, diversification of clades is restricted because species compete for limited resources. We hypothesize that such competition should not only affect numbers of species, but also prevent species from being phenotypically similar. Here, we present a method to detect whether competitive interactions between species have ordered phenotypic traits on a phylogeny, assuming that competition prevents related species from having identical trait values. We use the method to analyze clades of birds and mammals, with body size as the phenotypic trait. We find no sign that competition has prevented species from having the same body size. Thus, since body size is a key ecological trait and competition does not seem to be responsible for differences in body size between species, we conclude that the diversification slowdown that is prevalent in these clades is unlikely due to the ecological interference implied by the term density dependence.

## Introduction

A number of studies have recently investigated whether the branching times of reconstructed molecular phylogenies provide evidence of density-dependent speciation and/or extinction [1–5]. The term density-dependence stems from population ecology, where it has been used to describe the commonly observed phenomenon that per capita population growth decreases as the number of individuals in the population approaches the carrying capacity of its environment [6]. The species-level analogy is that the net rate of diversification of a clade decreases as the number of species increases [7–11].

Slowdown of diversification as species richness increases can have several causes (reviewed in [5]). One possibility is that the number of species is limited by resource availability in a fashion analogous to ecological density-dependence. However, there is an important distinction between density dependence in population ecology and diversification of species in clades. In ecological studies, individuals (i) occur at the same locality (ii) are phenotypically similar and therefore (iii) compete for a finite amount of resources. Resources cannot support a greater number of individuals because individuals cannot subdivide themselves into smaller units, and (generally) do not diverge enough phenotypically to be able to subsist on different resources. For evolutionary lineages, however, each of these three aspects may differ: (i) species may have spatially disjunct distributions, and (ii) they may diverge phenotypically so that (iii) they do not compete for the same resources. Moreover, the number of individuals per species can decrease, allowing available resources to support a greater total number of species. Thus, it is not clear that the dynamics of speciation and extinction would show density-dependence in such a way as to be analogous to density-dependent population growth.

It is difficult to detect evolutionary density-dependent diversification in nature because clades of species typically lack good fossil records (but see e.g. [12,13]). This is in opposition to ecological experiments where population size can be monitored continuously through time. Therefore, recent interest has focused on the detection of density-dependent diversification from molecular phylogenies of extant species [2–4]. Such methods, however, are analytically quite complex even for simple models of density-dependent diversification. Therefore, even the most sophisticated analyses of the branching times of molecular phylogenies assume that speciation and extinction rates depend on the (continental or even global) total number of species, and disregard factors like phenotypic divergence and geographic distribution that may ameliorate competition effects.

Given the difficulty of detecting density-dependent diversification from branching times of molecular phylogenies, it may be worthwhile to develop alternative methods testing whether competition for resources restricts the diversification of clades. Decades of ecological studies of phenotypically similar (but not necessarily closely related) species competing for resources indicate that competition is relaxed either by competitive exclusion or by character displacement i.e. the phenomenon where phenotypic differences between species become accentuated by competition for resources [14–17]. Current methods to detect density-dependent diversification from phylogenies focus on the evolutionary analogy of competitive exclusion, i.e. decreased net diversification (but see [18]). Here, our focus is instead on character displacement.

If diversification slowdown is due to processes analogous to ecological density-dependence, we expect not only signs of competitive exclusion (such as diversification slowdown), but also signs of character displacement on phylogenetic trees. Therefore, we develop a method to test whether character displacement has affected the order of trait values of extant species on a molecular phylogeny. First, we construct a conceptual model of how character displacement would affect trait evolution in a clade, and show the implications hereof for the patterning of trait values of the extant species on their phylogeny. We then use these findings to construct an algorithm to detect signs of character displacement in the traits of extant species. We illustrate the algorithm using body masses of North American continental *Dendroica* wood warblers, because this clade shows signs of density-dependent diversification [3,4]. Subsequently, we apply the algorithm to body sizes of extant bird and mammal species. We analyze body size because, as a correlate of many physiological and life history traits [19,20], body size is likely subject to character displacement [18,21]. Indeed, much of the evidence for character displacement has come from body size or strongly correlated traits [16,17,22,23] and frequently from mammals [24]. In addition, relatively well-resolved molecular phylogenies are available for several avian [25] and mammalian clades [26], most of which show signs of significant diversification slowdown (see below). Therefore, if this slowdown were due to processes equivalent to ecological density-dependence, we would expect to find ample evidence of character displacement on body size evolution in these groups of species.

## Methods

As a general model of character evolution, we will assume that the average trait values of evolving lineages change randomly over time. This would, for example, be the case under Brownian motion, Ornstein-Uhlenbeck, or punctuated equilibrium models of character evolution. In the absence of character displacement or limiting similarity, this null model would allow any two evolving lineages to have precisely the same trait value at any point in time. In our alternative model of character evolution, the trait of interest is subject to character displacement. Under this model, whenever two coexisting species develop similar average trait values, character displacement pushes them apart. Hence, two species will never have exactly the same average trait value.

In clades with exceptionally complete fossil records it may be possible to evaluate the effect of character displacement on trait evolution by counting in the fossil record how frequently species have had exactly the same trait value. Unfortunately, such complete fossil records are unavailable for most clades. From the phylogeny and trait values of existing species it is not possible to estimate the precise number of times that two species had the same trait value in history. However, it is possible to infer whether the extant species’ trait values could be achieved without any two species ever having exactly the same trait value.

**Fig 1:**
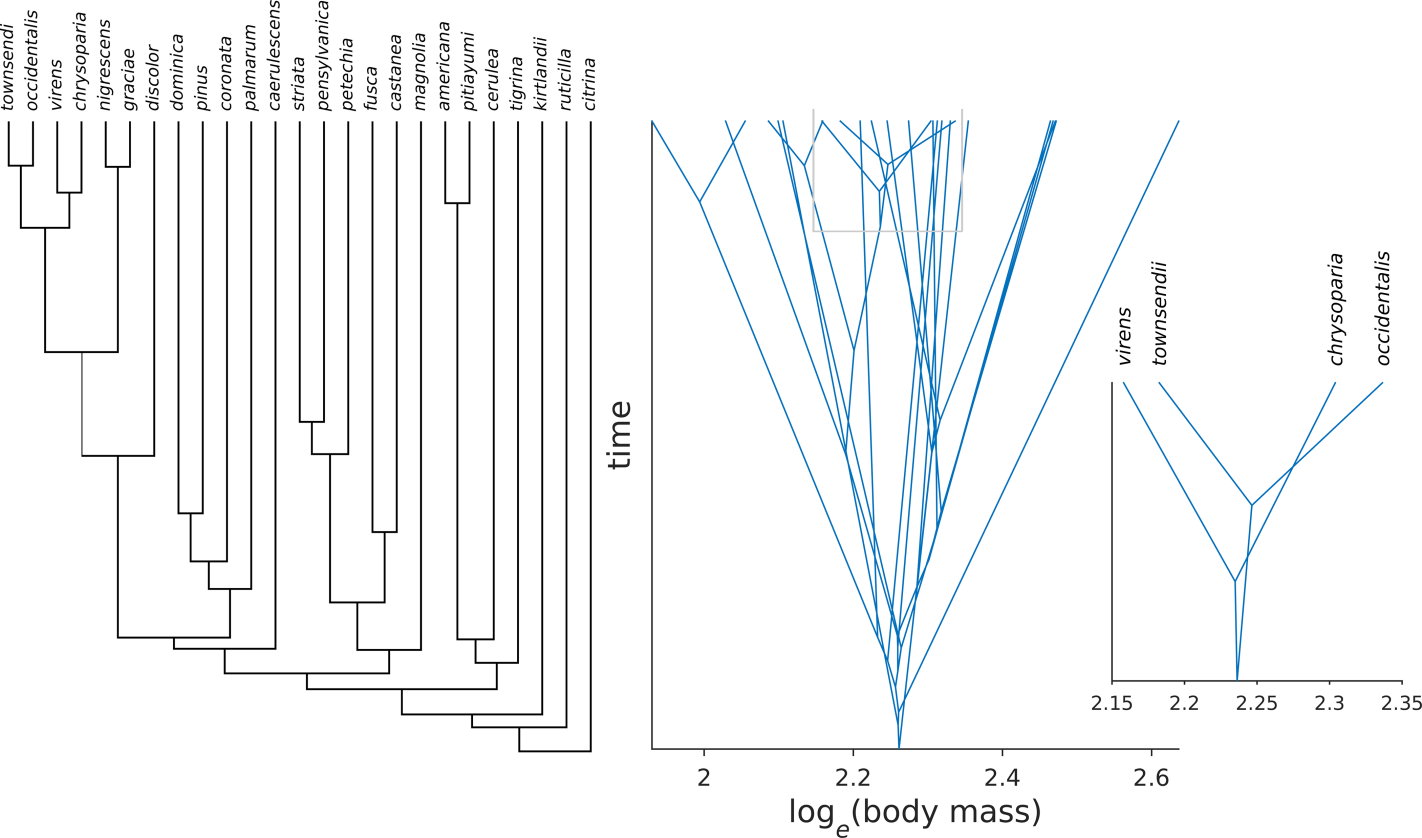
Phylogeny of continental North American *Dendroica* wood warblers, and a projection of body mass evolution on that phylogeny (middle), where body masses of present-day species are observed, and ancestral body masses inferred. The right panel zooms in on four closely related species: if the phylogeny is correct then the observed present-day body masses of *D*. *virens*, *D*. *townsendii*, *D*. *chrysoparia*, and *D*. *occidentalis* could not have evolved without some species having identical body masses at some point in time.

This is illustrated in Fig 1, which shows a molecular phylogeny of present-day *Dendroica* warblers [3] and their body masses [27]. The branching times of this phylogeny suggest a pattern of diversification slowdown, i.e. density dependence [3,4]. Ancestral body masses were inferred using maximum likelihood assuming a Brownian motion model of evolution. The right panel of Fig 1 shows the reconstructed body mass evolution of the species *D*. *virens*, *D*. *townsendii*, *D*. *chrysoparia*, and *D*. *occidentalis* and involves two line intersections, but it would be possible to draw the figure with only one intersection. For example, if the ancestor of *D*. *townsendii* and *D*. *occidentalis* is assigned a larger body mass, only the lines leading to *D*. *chrysoparia* and *D*. *townsendii* would intersect. However, it is not possible to connect the body masses of *D*. *virens*, *D*. *townsendii*, *D*. *chrysoparia*, and *D*. *occidentalis* on the molecular phylogeny so that there are no intersections. This means that some of their ancestors must have had exactly the same average body mass at least once over the course of their evolution. In other words, the present-day body mass configuration of *D*. *virens*, *D*. *townsendii*, *D*. *chrysoparia*, and *D*. *occidentalis* presents evidence against a model of character evolution in which character displacement prevents species from having the same trait value.

If the trait values of extant species could have evolved without any two species ever having the same trait value, then the trait values are perfectly ordered on the tree. For example in Fig 1, if we disregard *D*. *chrysoparia*, then the trait values of the species *D*. *virens*, *D*. *townsendii*, and *D*. *occidentalis* are ordered 1 2 3, and the tree topology can be written (*D*. *virens*,(*D*. *townsendii*, *D*. *occidentalis*)) in Newick format, which puts the species in the exact order of their trait values. If we add *D*. *chrysoparia*, the tree topology can no longer be written with the species in the order of body mass: we either get:

((*D*. *virens*, *D*. *chrysoparia)*, (*D*. *townsendii*, *D*. *occidentalis*)) or ((*D*. *townsendii*, *D*. *occidentalis*), (*D*. *virens*, *D*. *chrysoparia)*) but neither tree results in body mass order (i.e. 1 2 3 4). This simple example shows that the rank order of the extant species’ average trait values provides information about how frequently species have had identical trait values in the evolutionary history of the clade.

Inference of the frequency of different species having identical trait values from the rank order of trait values does (generally) not require a precise model of evolution under character displacement: it merely assumes that character displacement makes it unlikely that related species have had exactly the same average trait value at any point in time. We will use, as a statistic, *τ*: Kendall’s rank correlation between the species’ trait values and the position of the species in the Newick tree.

For the tree ((*D*. *virens*, *D*. *chrysoparia)*, (*D*. *townsendii*, *D*. *occidentalis*)), the rank correlation between the trait values and the position of the species in the tree is *τ* = 0.66. If we instead write ((*D*. *chrysoparia*, *D*. *virens)*, (*D*. *occidentalis*, *D*. *townsendii*)) the correlation is *τ* = 0. This shows that the rotation of a node affects the rank correlation between trait values and order of species in Newick notation. For larger trees with less ordered trait values (e.g. the 25 warblers in Fig 1), it will not be immediately apparent which Newick notation maximizes the rank correlation between trait values and species order. However, because of the definition of Kendall’s *τ*, the Newick notation that maximizes this correlation can be found using an iterative node rotation algorithm: starting from the most recent node in the tree, each node is rotated once (so that sister species change places) and the correlation of the trait values of the species descending from this node is re-calculated. If the rank correlation for this node rotation produces a higher correlation than the previous calculation, then the rotation is accepted and the algorithm continues to the next most recent node. Using this algorithm, if the maximized rank correlation is high (i.e. close to one) then the trait order on the tree has likely been affected by character displacement, and if there has been no such bias then the rank correlation is expected to be closer to zero.

**Fig 2:**
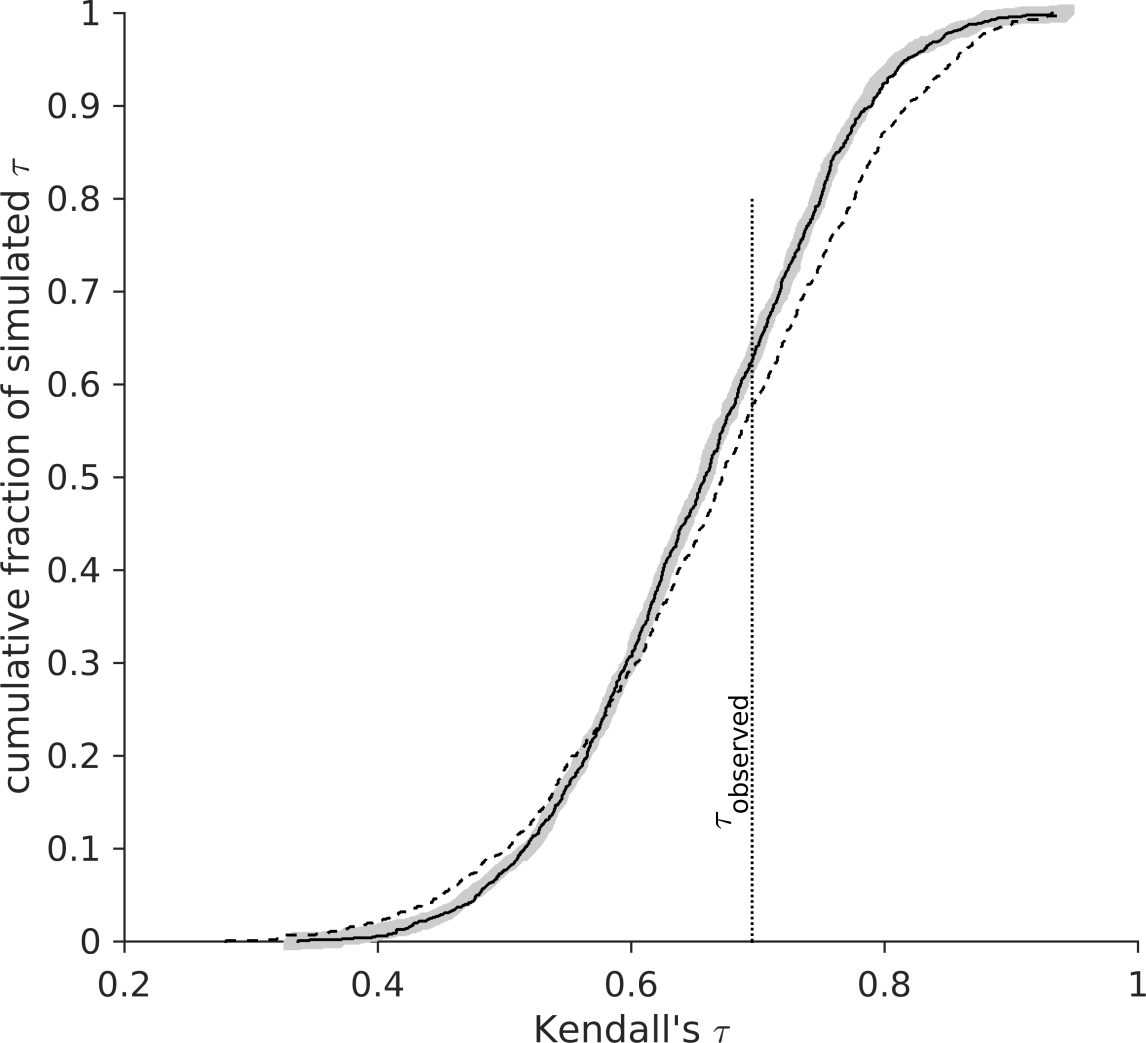
A: Distribution of rank correlations (Kendall’s *τ*) for trait values randomly simulated on the *Dendroica* phylogeny with 10-fold different rates of evolution (thick grey and narrow black solid lines). The distributions of 1000 *τ* each are not significantly different. Dashed line: Distribution of *τ* for trait values randomly simulated on a phylogeny with equal number of tip species, but different topology than the *Dendroica* phylogeny. Dotted vertical line indicates the value of *τ* obtained with the observed body masses of present-day *Dendroica* warblers.

In order to evaluate whether trait values of species are more ordered than expected under random phenotypic evolution, we must determine the sampling distribution of *τ*. This distribution does not depend on the rate of evolution: a high rate of evolution simply scales trait space; hence it leads to greater interspecific variance, but not to more crossing branches (Fig 2). Instead, the sampling distribution of *τ* is determined by the topology, branching times, and (obviously) the number of branches of the phylogeny (Fig 2). Thus, to estimate the probability *p* that the observed order of species’ trait values on the phylogeny is the result of unbiased evolution we compare *τ*_*obs*_ calculated from the observed trait values to *τ*_*sim*_ calculated from trait values simulated *s* times on the observed phylogeny. If *τ*_*sim*_ > τ_obs_, in s* out of *s* simulations, *p* = (s*+1)/(*s*+1). (This test is one-sided, because it is based on correlations that were maximized by node rotation.) For the example of *Dendroica*, *p* = 0.63 (Fig 2), suggesting the pattern of diversification slowdown in this clade [3,4] is due to something other than ecological competition leading to character displacement potentially resulting in density-dependence.

To demonstrate our approach, we apply it to extant avian and mammalian body masses (data from [27] and [28] respectively) using relatively well-resolved global phylogenetic trees [25,26] to investigate whether there are any signs of character displacement that suggest that diversification slowdown could be driven by ecological density dependence. Clades were extracted using the following criteria: the number of species must be greater than 10 (for statistical power); the crown age of a clade must not exceed 15my in order to compare relatively closely related species; trait data, in our case body size, should be available for at least 80% of species. For each clade, we simulated unbiased body size evolution 1000 times on each phylogeny assuming Brownian motion, to determine the sampling distribution of *τ* under the null model, in order to calculate *p*-values as explained above. In addition, we calculated the *γ*-statistic [29] to infer whether the phylogeny analyzed likely experienced diversification slowdown over time.

## Results

The majority of avian and mammalian clades that we analyzed showed signs of diversification slowdown: 114 out of 140 avian and 14 out of 19 mammalian clades had negative γ. (γ<0 indicates a decrease in the rate of lineage accumulation over time as compared to the expectation under a constant-rates speciation-extinction process.) However, none of the clades showed significant (*α≤*0.05) trait order (high *τ*) after correction for multiple testing using both the conservative Bonferroni correction and less conservative, False Discovery Rate (FDR) [30,31] (S1 Table). There was no tendency for clades with strong signs of density-dependent diversification (low values of *γ*) to show body mass order on the phylogeny (high *τ*). In birds, the correlation between *γ* and *τ* was positive, in mammals, the correlation was negative but not statistically significant.

## Discussion

We present a new, general method that can be used to infer whether character displacement, or similar processes, affected phylogenetic trait order, using only trait values and a phylogeny of present-day species. The method uses the ranks of trait values, and is therefore relatively insensitive to the particular model of evolution under character displacement: it merely assumes that character displacement makes it unlikely that related species have had identical trait values. However, because branch lengths affect the sampling distribution of *τ* (Fig 2), so does the model of character evolution (e.g. Brownian motion or Ornstein-Uhlenbeck). Therefore, in case of borderline significant results, a realistic model of evolution should be used, and phylogenetic uncertainty accounted for.

Application of this method to clades of birds and mammals did not reveal any sign of character displacement affecting long-term body size evolution. These results are not due to a lack of statistical power: In the absence of character displacement, *τ* = 1 is observed in only 2% of cases for a phylogeny of as few as 10 species, and almost never for a phylogeny of 25 or more species (Fig 2). That is because the number of branch crossings increases faster than linearly with the number of tip species: a phylogeny with 10 tip species has on average about 10 branch crossings, but a phylogeny of 100 species has, on average, more than 700 branch crossings! Hence, statistical power of the present method in combination with the data analyzed here is sufficiently high if character displacement is strong.

Ultimately the statistical power of our test depends not on the number of species, but on the strength of character displacement (or more generally, phenotypic order on the tree). In the framework of the method we present here, we can model the strength of character displacement as the probability *p*_*c*_ that two species have exactly the same character state. We assume *p*_*c*_ = 0, but the weaker the effect character displacement has on trait evolution, the larger *p*_*c*_ becomes. If *p*_*c*_ = 1, character displacement is absent and does not affect trait evolution. Hence, it could be argued that the lack of evidence for character displacement influencing long-term evolution of body size reflects only weak character displacement. However, even with weak character displacement (e.g. *p*_*c*_ = 0.5), we still expect trait values to show substantial order especially on larger phylogenies. That is because the order of traits (*τ*) is negatively related to the number of crossing branches. For a phylogeny with 25 tip species (Fig 1), the number of crossing branches is, on average, 44. Even if branch crossings occur with probability *p*_*c*_ = 0.5, the probability to observe a tree with 44 crossings is 0.5^44^≈0. Obviously, plausible trees have only a tiny fraction of the number of branch crossings that would be expected under unbiased evolution (*p*_*c*_ = 1). In other words, even under weak character displacement, expected numbers of crossing branches are far smaller than under unbiased evolution and hence, order of trait values (*τ*) is far greater than under unbiased evolution, especially in larger phylogenies. Moreover, body size is a crucial ecological trait and the prime example of a trait subject to limiting similarity and character displacement on ecological time scales [24]. Therefore, it seems unlikely that the negative results of our analyses are due to low statistical power, or weak character displacement.

It may be argued, that our results are biased towards a lack of character displacement because we use extant species data [32,33] thus, the effects of extinction are muted [12]. We argue that by using a phylogeny of related species our analysis captures historical changes in characters over time so long as these historical interactions have resulted in changes in present-day character evolution. That being said, the analyses presented here, have limited power to detect signs of historical processes such as competition involving species that are now extinct [32,33] and therefore have been pruned from the phylogeny. This is a limitation of the data, not of the method we propose. If historical trait data (for species that are now extinct) is available, and it is possible to accurately place the data into the phylogeny, one could use our method to more fully investigate the effects that historical traits and species interactions have had on trait evolution over time.

A different, yet probable explanation for our negative results is that the species in the clades we studied have (had) disjunct global or local distributions (e.g. different habitat use [34]) so that body size evolution is not the result of competition. A recent study suggests that many phenotypic differences between lineages of ovenbirds are due to the amount of time lineages have been separated, not to species distributions or potential interactions [35]. We ran additional analyses where we added the criterion that a clade should not only have a minimum of 10 species and a crown age less than 15 my, but also include at least 10 species with mutually overlapping geographic distributions. (Note: this does not imply that there is some location where all 10 species co-occur, but that each species geographically overlaps with at least one other species in the clade.) If competition would cause patterning of body mass on the phylogeny, such clades would show high values of *τ*. However, using geographic range data from the International Union for Conservation of Nature and Natural Resources [36] we found no such clades, even though there are many clades with crown ages less than 15my and many sets of 10 or more mutually geographically overlapping species. This implies that closely related species, which are often ecologically similar, tend to have disjunct distributions.

Over millions of years phenotypic and ecological differences accumulate; thus, it becomes more likely that similar species coexist. Identical body sizes may occur if species differ substantially in other traits (e.g. beak shape [17] or tooth length [22]). Similarly, selective pressures change over time, and more than one pressure may act on a specific trait [37,38]. Thus, unless competition produces constant, strong directional trait change, or after divergence there is no other pressure acting upon body size, the evolutionary patters we expect to find may be muted or disappear completely (this is suggested for traits where sexual selection is thought to drive differentiation between species. This strong, directional selection is suggested to have a prominent effect during the initial stages of speciation/trait evolution but subsequently is muted or erased by other selective pressures [39]). On a time scale that spans migration, speciation, and extinction, as species shift distributions and diversify, it seems unlikely that one selective pressure would remain so dominant as to leave a signal spanning longer than the initial event leading to diversification between genetically similar species. Thus, although it is possible that competition effects body size, competition may not maintain these differences or be responsible for further changes in body size over time. Therefore, it seems unlikely that diversification slowdown in these lineages is due to some evolutionary equivalent of ecological density-dependence.

Slowdown in the rate of lineage accumulation over time has been observed in fossil records [4, 40–42] as well as molecular phylogenies of clades of animals [1,2,4] and plants [43]. Such slowdown could be due to ecological density-dependence, but also to other causes such as adaptive radiation [2,44], protracted speciation [45], incorrect or incomplete phylogenies [41,46,47], niche filling, or other causes [5]. Indeed, there is very little direct evidence that decelerating diversification rates are caused by increasing global numbers of species. Therefore, the term density- or diversity- dependence should not be used indiscriminately when diversification slows down, but only when there is evidence of causal density-dependence. Ideally, to distinguish density-dependence from alternative explanations, future analyses of diversification slowdown should take species geographic distribution [42], 2016) and phenotypic differences [22,48] into account [5], and not rely solely on the global numbers of species in a clade.

## Supporting information

S1 Table Individual clades analyzed. Clades are ordered by *p*-values. One-sided *p*-values were determined from 1000 simulations of unbiased body size evolution on the phylogenetic tree of each clade: *p* = (s*+1)/(1001). To be significant at the 5% level after Bonferroni correction, a bird clade would need an uncorrected *p* ≤ 0.00036; and a mammal clade *p* ≤ 0.0027. Adjusted *p*-values for both the Bonferroni correction and the False Discovery Rate (FDR) are presented for each clade.
(DOCX)

## References

[1] Nee S, Mooers AO, Harvey PH (1992) Tempo and mode of evolution revealed from molecular phylogenies. Proc Natl Acad Sci U S A 89: 8322–8326. 1518865 10.1073/pnas.89.17.8322 PMC49910

[2] Phillimore AB, Price TD (2008) Density-dependent cladogenesis in birds. PLoS Biol 6: e71 doi: 10.1371/journal.pbio.0060071 18366256 10.1371/journal.pbio.0060071 PMC2270327

[3] Rabosky DL, Lovette IJ (2008) Explosive evolutionary radiations: decreasing speciation or increasing extinction through time? Evolution 62: 1866–1875. doi: 10.1111/j.1558-5646.2008.00409.x 18452577 10.1111/j.1558-5646.2008.00409.x

[4] Etienne RS, Haegeman B, Stadler T, Aze T, Pearson PN, et al (2012) Diversity-dependence brings molecular phylogenies closer to agreement with the fossil record. Proc Biol Sci 279: 1300–1309. doi: 10.1098/rspb.2011.1439 21993508 10.1098/rspb.2011.1439 PMC3282358

[5] Moen D, Morlon H (2014) Why does diversification slow down? Trends Ecol Evol (Amst) 29: 190–197. doi: 10.1016/j.tree.2014.01.010 24612774 10.1016/j.tree.2014.01.010

[6] Turchin P (1999) Population Regulation: A Synthetic View. Oikos 84: 153 doi: 10.2307/3546876

[7] Stanley S (1973) Effectsof competition on rates of evolution, with special references to bivalve mollusks and mammals. Syst Zool 22: 486–506.

[8] Gould S, Raup D, Sepkoski J, Schopf T, Simberloff D (1977) The shape of evolution: a comparison of real and random clades. Paleobiology 3: 23–40.

[9] Cornell HV (2013) Is regional species diversity bounded or unbounded? Biol Rev Camb Philos Soc 88: 140–165. doi: 10.1111/j.1469-185X.2012.00245.x 22958676 10.1111/j.1469-185X.2012.00245.x

[10] Rabosky DL (2013) Diversity-Dependence, Ecological Speciation, and the Role of Competition in Macroevolution. Annu Rev Ecol Evol Syst 44: 481–502. doi: 10.1146/annurev-ecolsys-110512-135800

[11] Harmon LJ, Harrison S (2015) Species diversity is dynamic and unbounded at local and continental scales. Am Nat 185: 584–593. doi: 10.1086/680859 25905502 10.1086/680859

[12] Ezard THG, Aze T, Pearson PN, Purvis A (2011) Interplay between changing climate and species’ ecology drives macroevolutionary dynamics. Science 332: 349–351. doi: 10.1126/science.1203060 21493859 10.1126/science.1203060

[13] Silvestro D, Antonelli A, Salamin N, Quental TB (2015) The role of clade competition in the diversification of North American canids. Proc Natl Acad Sci U S A 112: 8684–8689. doi: 10.1073/pnas.1502803112 26124128 10.1073/pnas.1502803112 PMC4507235

[14] Brown W, Wilson E (1956) Character displacement. Syst Zool 5: 49–64.

[15] Abrams P (1983) The Theory of Limiting Similarity. Annu Rev Ecol Syst 14: 359–376. doi: 10.1146/annurev.es.14.110183.002043

[16] Schluter D (2000) Ecological Character Displacement in Adaptive Radiation. Am Nat 156: S4–S16. doi: 10.1086/303412

[17] Grant P, Grant B (2006) Evolution of character displacement in Darwin’s finches. Science 313: 224–226. doi: 10.1126/science.1128374 16840700 10.1126/science.1128374

[18] Davies TJ, Cooper N, Diniz-Filho JAF, Thomas GH, Meiri S (2012) Using phylogenetic trees to test for character displacement: a model and an example from a desert mammal community. Ecology 93: S44–S51. doi: 10.1890/11-0400.1

[19] Calder W (1984) Size, function, and life history. Mineola, NY, USA: Dover Publications.

[20] Schmidt-Nielsen K (1984) Scaling: Why is animal size so important Cambridge, UK: Cambridge University Press.

[21] Dayan T, Simberloff D (2005) Ecological and community-wide character displacement: the next generation. Ecol Lett 8: 875–894. doi: 10.1111/j.1461-0248.2005.00791.x

[22] Davies TJ, Meiri S, Barraclough TG, Gittleman JL (2007) Species co-existence and character divergence across carnivores. Ecol Lett 10: 146–152. doi: 10.1111/j.1461-0248.2006.01005.x 17257102 10.1111/j.1461-0248.2006.01005.x

[23] Reifová R, Reif J, Antczak M, Nachman MW (2011) Ecological character displacement in the face of gene flow: evidence from two species of nightingales. BMC Evol Biol 11: 138 doi: 10.1186/1471-2148-11-138 21609448 10.1186/1471-2148-11-138 PMC3121626

[24] Dayan T, Simberloff D (1998) Size patterns among competitors: ecological character displacement and character release in mammals, with special reference to island populations. Mammal review 28: 99–124.

[25] Jetz W, Thomas GH, Joy JB, Redding DW, Hartmann K, et al (2014) Global distribution and conservation of evolutionary distinctness in birds. Curr Biol 24: 919–930. doi: 10.1016/j.cub.2014.03.011 24726155 10.1016/j.cub.2014.03.011

[26] Martyn I, Kuhn TS, Mooers AO, Moulton V, Spillner A (2012) Computing evolutionary distinctiveness indices in large scale analysis. Algorithms Mol Biol 7: 6 doi: 10.1186/1748-7188-7-6 22502588 10.1186/1748-7188-7-6 PMC3353162

[27] Dunning J (1993) CRC handbook of avian body masses. Boca Raton: CRC Press.

[28] Smith F, Lyons S, Ernest S, Jones K, Kaufman D, et al (2003) Body mass of late Quaternary mammals. Ecology 84: 3402.

[29] Pybus OG, Harvey PH (2000) Testing macro-evolutionary models using incomplete molecular phylogenies. Proc Biol Sci 267: 2267–2272. doi: 10.1098/rspb.2000.1278 11413642

[30] Benjamini Y, Hochberg Y (n.d.) Controlling the False Discovery Rate: A Practical and Powerful Approach to Multiple Testing.

[31] Narum SR (2006) Beyond Bonferroni: Less conservative analyses for conservation genetics. Conserv Genet 7: 783–787. doi: 10.1007/s10592-005-9056-y

[32] Carotenuto F, Barbera C, Raia P (2010) Occupancy, range size, and phylogeny in Eurasian Pliocene to Recent large mammals. Paleobiology 36: 399–414. doi: 10.1666/09059.1

[33] Carotenuto F, Diniz-Filho JAF, Raia P (2015) Space and time: The two dimensions of Artiodactyla body mass evolution. Palaeogeography, Palaeoclimatology, Palaeoecology 437: 18–25. doi: 10.1016/j.palaeo.2015.07.013

[34] Connell JH (1980) Diversity and the Coevolution of Competitors, or the Ghost of Competition Past. Oikos 35: 131 doi: 10.2307/3544421

[35] Tobias JA, Cornwallis CK, Derryberry EP, Claramunt S, Brumfield RT, et al (2014) Species coexistence and the dynamics of phenotypic evolution in adaptive radiation. Nature 506: 359–363. doi: 10.1038/nature12874 24362572 10.1038/nature12874

[36] IUCN Red List of Threatened Species (2009). Available: http://www.iucn-redlist.org.

[37] Darwin C (1859) On the Origin of Species by Means of Natural Selection, or the Preservation of Favoured Races in the Struggle for Life. London, UK: John Murray Available: http://darwin-online.org.uk/converted/pdf/1861_OriginNY_F382.pdf. PMC5184128 30164232

[38] Arnqvist G, Rowe L (2005) Sexual Conflict. New Jersey, USA: Princeton University Press.

[39] Kraaijeveld K, Kraaijeveld-Smit FJL, Maan ME (2011) Sexual selection and speciation: the comparative evidence revisited. Biol Rev Camb Philos Soc 86: 367–377. doi: 10.1111/j.1469-185X.2010.00150.x 20659104 10.1111/j.1469-185X.2010.00150.x

[40] Benton MJ, Emerson BC (2007) How did life become so diverse? the dynamics of diversification according to the fossil record and molecular phylogenetics. Palaeontology 50: 23–40. doi: 10.1111/j.1475-4983.2006.00612.x

[41] Quental TB, Marshall CR (2010) Diversity dynamics: molecular phylogenies need the fossil record. Trends Ecol Evol (Amst) 25: 434–441. doi: 10.1016/j.tree.2010.05.002 20646780 10.1016/j.tree.2010.05.002

[42] Raia P, Carotenuto F, Mondanaro A, Castiglione S, Passaro F, et al (2016) Progress to extinction: increased specialisation causes the demise of animal clades. Sci Rep 6: 30965 doi: 10.1038/srep30965 27507121 10.1038/srep30965 PMC4978992

[43] McPeek MA (2008) The ecological dynamics of clade diversification and community assembly. Am Nat 172: E270–84. doi: 10.1086/593137 18851684 10.1086/593137

[44] Harmon LJ, Schulte JA, Larson A, Losos JB (2003) Tempo and mode of evolutionary radiation in iguanian lizards. Science 301: 961–964. doi: 10.1126/science.1084786 12920297 10.1126/science.1084786

[45] Etienne RS, Rosindell J (2012) Prolonging the past counteracts the pull of the present: protracted speciation can explain observed slowdowns in diversification. Syst Biol 61: 204–213. doi: 10.1093/sysbio/syr091 21873376 10.1093/sysbio/syr091 PMC3280041

[46] Revell LJ, Harmon LJ, Glor RE (2005) Underparameterized model of sequence evolution leads to bias in the estimation of diversification rates from molecular phylogenies. Syst Biol 54: 973–983. 16385778 10.1080/10635150500354647

[47] Cusimano N, Renner SS (2010) Slowdowns in diversification rates from real phylogenies may not be real. Syst Biol 59: 458–464. doi: 10.1093/sysbio/syq032 20547781 10.1093/sysbio/syq032

[48] Cooper N, Purvis A (2009) What factors shape rates of phenotypic evolution? A comparative study of cranial morphology of four mammalian clades. J Evol Biol 22: 1024–1035. doi: 10.1111/j.1420-9101.2009.01714.x 21462402 10.1111/j.1420-9101.2009.01714.x

