# Supplementary material for: Does density-dependent diversification mirror ecological competitive exclusion?
Source: PLoS One. 2017 Oct 12;12(10):e0184814. doi: 10.1371/journal.pone.0184814 (PMC5638247; doi:10.1371/journal.pone.0184814)
Supplement: S1 Table — Clades are ordered by p-values. One-sided p-values were determined from 1000 simulations of unbiased body size evolution on the phylogenetic tree of each clade: p = (s*+1)/(1001). To be significant at the 5% level after Bonferroni correction, a bird clade would need an uncorrected p ≤ 0.00036; and a mammal clade p ≤ 0.0027. Adjusted p-values for both the Bonferroni correction and the False Discovery Rate (FDR) are presented for each clade. (DOCX) [file pone.0184814.s001.docx]

|  | **No. species in clade** | **No. species with mass data** | **Crown age** | ***γ*** | ***τ_obs_*** | ***p-value*** | **Bonferroni corrected *p-value*** | **FDR corrected *p-value*** |
| --- | --- | --- | --- | --- | --- | --- | --- | --- |
| **Birds** | 15 | 13 | 12.906 | -0.245 | 0.974 | 0.002 | 0.280 | 0.140 |
|  | 263 | 214 | 14.745 | -6.174 | 0.676 | 0.002 | 0.280 | 0.140 |
|  | 16 | 15 | 12.060 | -2.766 | 0.924 | 0.003 | 0.420 | 0.140 |
|  | 189 | 164 | 14.760 | -2.945 | 0.672 | 0.023 | 1.000 | 0.804 |
|  | 12 | 10 | 13.148 | -0.609 | 0.956 | 0.040 | 1.000 | 0.956 |
|  | 43 | 37 | 12.323 | -2.578 | 0.718 | 0.041 | 1.000 | 0.956 |
|  | 46 | 37 | 14.524 | -4.166 | 0.664 | 0.059 | 1.000 | 1.000 |
|  | 374 | 324 | 12.994 | -5.312 | 0.468 | 0.073 | 1.000 | 1.000 |
|  | 39 | 33 | 13.782 | -3.221 | 0.602 | 0.078 | 1.000 | 1.000 |
|  | 22 | 18 | 13.992 | -0.067 | 0.791 | 0.081 | 1.000 | 1.000 |
|  | 12 | 11 | 11.024 | 0.708 | 0.891 | 0.128 | 1.000 | 1.000 |
|  | 18 | 16 | 14.115 | -2.853 | 0.717 | 0.136 | 1.000 | 1.000 |
|  | 41 | 37 | 14.084 | -0.338 | 0.646 | 0.142 | 1.000 | 1.000 |
|  | 32 | 31 | 14.303 | -0.483 | 0.669 | 0.174 | 1.000 | 1.000 |
|  | 33 | 28 | 13.798 | -1.658 | 0.566 | 0.187 | 1.000 | 1.000 |
|  | 11 | 10 | 8.026 | -1.186 | 0.778 | 0.203 | 1.000 | 1.000 |
|  | 44 | 42 | 11.873 | -2.183 | 0.617 | 0.206 | 1.000 | 1.000 |
|  | 33 | 30 | 14.680 | -1.503 | 0.559 | 0.217 | 1.000 | 1.000 |
|  | 10 | 8 | 11.935 | -0.664 | 0.857 | 0.226 | 1.000 | 1.000 |
|  | 15 | 13 | 14.065 | -2.471 | 0.641 | 0.231 | 1.000 | 1.000 |
|  | 39 | 36 | 13.406 | -2.489 | 0.619 | 0.235 | 1.000 | 1.000 |
|  | 21 | 19 | 14.335 | -0.413 | 0.684 | 0.239 | 1.000 | 1.000 |
|  | 25 | 22 | 14.886 | -1.475 | 0.636 | 0.245 | 1.000 | 1.000 |
|  | 25 | 25 | 10.550 | -2.613 | 0.607 | 0.245 | 1.000 | 1.000 |
|  | 25 | 23 | 13.482 | 2.068 | 0.731 | 0.267 | 1.000 | 1.000 |
|  | 14 | 12 | 14.234 | -0.209 | 0.727 | 0.277 | 1.000 | 1.000 |
|  | 29 | 28 | 14.426 | -0.721 | 0.614 | 0.285 | 1.000 | 1.000 |
|  | 11 | 9 | 9.949 | 1.204 | 0.889 | 0.287 | 1.000 | 1.000 |
|  | 12 | 11 | 11.571 | 0.558 | 0.818 | 0.293 | 1.000 | 1.000 |
|  | 10 | 9 | 12.152 | 0.364 | 0.833 | 0.294 | 1.000 | 1.000 |
|  | 52 | 50 | 8.336 | 1.500 | 0.629 | 0.312 | 1.000 | 1.000 |
|  | 22 | 18 | 9.380 | -2.938 | 0.621 | 0.330 | 1.000 | 1.000 |
|  | 11 | 10 | 14.452 | -1.128 | 0.733 | 0.345 | 1.000 | 1.000 |
|  | 20 | 17 | 11.143 | -2.030 | 0.706 | 0.353 | 1.000 | 1.000 |
|  | 15 | 13 | 12.675 | -1.995 | 0.615 | 0.369 | 1.000 | 1.000 |
|  | 21 | 20 | 13.998 | -2.050 | 0.579 | 0.369 | 1.000 | 1.000 |
|  | 39 | 38 | 14.915 | -1.243 | 0.548 | 0.376 | 1.000 | 1.000 |
|  | 10 | 10 | 14.849 | -0.889 | 0.733 | 0.388 | 1.000 | 1.000 |
|  | 20 | 17 | 13.891 | -0.476 | 0.647 | 0.399 | 1.000 | 1.000 |
|  | 13 | 11 | 10.072 | -2.087 | 0.709 | 0.405 | 1.000 | 1.000 |
|  | 17 | 14 | 11.860 | -0.750 | 0.670 | 0.405 | 1.000 | 1.000 |
|  | 22 | 18 | 12.788 | -2.465 | 0.595 | 0.406 | 1.000 | 1.000 |
|  | 14 | 13 | 11.908 | -1.614 | 0.641 | 0.417 | 1.000 | 1.000 |
|  | 32 | 28 | 11.310 | -0.409 | 0.587 | 0.424 | 1.000 | 1.000 |
|  | 12 | 11 | 14.407 | -0.565 | 0.745 | 0.428 | 1.000 | 1.000 |
|  | 109 | 108 | 8.238 | 0.515 | 0.465 | 0.428 | 1.000 | 1.000 |
|  | 11 | 10 | 13.212 | 0.153 | 0.778 | 0.432 | 1.000 | 1.000 |
|  | 15 | 13 | 10.529 | -2.080 | 0.615 | 0.439 | 1.000 | 1.000 |
|  | 10 | 8 | 14.041 | -0.090 | 0.714 | 0.450 | 1.000 | 1.000 |
|  | 10 | 10 | 13.307 | -1.098 | 0.689 | 0.457 | 1.000 | 1.000 |
|  | 13 | 13 | 13.135 | -2.167 | 0.538 | 0.471 | 1.000 | 1.000 |
|  | 32 | 27 | 14.384 | -2.303 | 0.499 | 0.476 | 1.000 | 1.000 |
|  | 71 | 68 | 14.044 | -0.929 | 0.507 | 0.476 | 1.000 | 1.000 |
|  | 10 | 9 | 13.163 | 1.292 | 0.778 | 0.479 | 1.000 | 1.000 |
|  | 10 | 8 | 14.454 | -0.336 | 0.786 | 0.481 | 1.000 | 1.000 |
|  | 14 | 14 | 13.797 | -1.551 | 0.560 | 0.501 | 1.000 | 1.000 |
|  | 16 | 13 | 13.880 | -0.096 | 0.641 | 0.510 | 1.000 | 1.000 |
|  | 10 | 8 | 7.192 | -0.811 | 0.714 | 0.530 | 1.000 | 1.000 |
|  | 28 | 24 | 14.135 | -1.133 | 0.507 | 0.536 | 1.000 | 1.000 |
|  | 28 | 24 | 14.122 | -1.483 | 0.529 | 0.538 | 1.000 | 1.000 |
|  | 142 | 123 | 13.334 | -2.731 | 0.357 | 0.576 | 1.000 | 1.000 |
|  | 35 | 30 | 14.329 | -0.794 | 0.568 | 0.581 | 1.000 | 1.000 |
|  | 19 | 18 | 6.147 | 0.202 | 0.621 | 0.588 | 1.000 | 1.000 |
|  | 14 | 14 | 13.820 | -1.709 | 0.538 | 0.592 | 1.000 | 1.000 |
|  | 12 | 11 | 13.513 | -0.102 | 0.636 | 0.602 | 1.000 | 1.000 |
|  | 29 | 26 | 14.758 | -1.175 | 0.545 | 0.605 | 1.000 | 1.000 |
|  | 10 | 10 | 12.322 | -1.415 | 0.600 | 0.612 | 1.000 | 1.000 |
|  | 12 | 12 | 9.915 | -0.291 | 0.606 | 0.617 | 1.000 | 1.000 |
|  | 23 | 22 | 14.268 | 0.245 | 0.567 | 0.630 | 1.000 | 1.000 |
|  | 19 | 19 | 12.388 | -1.099 | 0.509 | 0.646 | 1.000 | 1.000 |
|  | 99 | 84 | 14.898 | -4.964 | 0.383 | 0.666 | 1.000 | 1.000 |
|  | 17 | 14 | 14.646 | -1.581 | 0.473 | 0.684 | 1.000 | 1.000 |
|  | 24 | 22 | 9.632 | -2.226 | 0.498 | 0.693 | 1.000 | 1.000 |
|  | 18 | 17 | 14.030 | -0.352 | 0.500 | 0.704 | 1.000 | 1.000 |
|  | 12 | 11 | 11.895 | 0.437 | 0.564 | 0.711 | 1.000 | 1.000 |
|  | 16 | 15 | 13.208 | -0.568 | 0.562 | 0.712 | 1.000 | 1.000 |
|  | 18 | 16 | 13.331 | -1.726 | 0.500 | 0.719 | 1.000 | 1.000 |
|  | 12 | 11 | 14.413 | -1.008 | 0.527 | 0.721 | 1.000 | 1.000 |
|  | 11 | 11 | 12.715 | 0.696 | 0.600 | 0.734 | 1.000 | 1.000 |
|  | 36 | 33 | 14.050 | -0.608 | 0.500 | 0.742 | 1.000 | 1.000 |
|  | 14 | 14 | 11.716 | -2.358 | 0.473 | 0.764 | 1.000 | 1.000 |
|  | 40 | 33 | 14.512 | -1.319 | 0.458 | 0.767 | 1.000 | 1.000 |
|  | 20 | 17 | 13.698 | -1.231 | 0.426 | 0.778 | 1.000 | 1.000 |
|  | 115 | 99 | 13.928 | -1.173 | 0.370 | 0.783 | 1.000 | 1.000 |
|  | 12 | 12 | 14.789 | 0.957 | 0.515 | 0.787 | 1.000 | 1.000 |
|  | 10 | 10 | 10.592 | -1.357 | 0.556 | 0.797 | 1.000 | 1.000 |
|  | 12 | 10 | 13.887 | 0.962 | 0.644 | 0.813 | 1.000 | 1.000 |
|  | 17 | 15 | 12.210 | -1.348 | 0.467 | 0.814 | 1.000 | 1.000 |
|  | 10 | 9 | 13.377 | -0.362 | 0.611 | 0.821 | 1.000 | 1.000 |
|  | 18 | 16 | 12.222 | -1.501 | 0.500 | 0.826 | 1.000 | 1.000 |
|  | 13 | 12 | 9.915 | -0.247 | 0.515 | 0.842 | 1.000 | 1.000 |
|  | 19 | 19 | 14.019 | -1.338 | 0.380 | 0.844 | 1.000 | 1.000 |
|  | 10 | 8 | 14.486 | -1.195 | 0.500 | 0.845 | 1.000 | 1.000 |
|  | 13 | 11 | 7.106 | -1.789 | 0.455 | 0.847 | 1.000 | 1.000 |
|  | 13 | 12 | 13.418 | 0.136 | 0.485 | 0.858 | 1.000 | 1.000 |
|  | 14 | 13 | 13.395 | -0.827 | 0.436 | 0.874 | 1.000 | 1.000 |
|  | 11 | 10 | 8.461 | 0.088 | 0.511 | 0.893 | 1.000 | 1.000 |
|  | 17 | 15 | 12.287 | -0.883 | 0.505 | 0.896 | 1.000 | 1.000 |
|  | 17 | 16 | 12.655 | -0.692 | 0.450 | 0.899 | 1.000 | 1.000 |
|  | 31 | 25 | 12.033 | -2.695 | 0.367 | 0.902 | 1.000 | 1.000 |
|  | 50 | 43 | 10.977 | -0.476 | 0.364 | 0.904 | 1.000 | 1.000 |
|  | 15 | 15 | 12.204 | -3.163 | 0.371 | 0.906 | 1.000 | 1.000 |
|  | 11 | 11 | 10.909 | -2.201 | 0.418 | 0.910 | 1.000 | 1.000 |
|  | 121 | 98 | 14.876 | 0.041 | 0.341 | 0.910 | 1.000 | 1.000 |
|  | 10 | 8 | 12.524 | 0.908 | 0.643 | 0.913 | 1.000 | 1.000 |
|  | 14 | 12 | 13.389 | -2.005 | 0.364 | 0.914 | 1.000 | 1.000 |
|  | 51 | 44 | 13.900 | -0.917 | 0.338 | 0.922 | 1.000 | 1.000 |
|  | 11 | 11 | 11.426 | -0.036 | 0.418 | 0.929 | 1.000 | 1.000 |
|  | 17 | 15 | 13.059 | 0.187 | 0.371 | 0.931 | 1.000 | 1.000 |
|  | 24 | 21 | 14.329 | -2.426 | 0.333 | 0.938 | 1.000 | 1.000 |
|  | 34 | 32 | 14.026 | -2.256 | 0.302 | 0.949 | 1.000 | 1.000 |
|  | 30 | 24 | 13.911 | 0.931 | 0.348 | 0.950 | 1.000 | 1.000 |
|  | 11 | 9 | 11.986 | -1.622 | 0.389 | 0.951 | 1.000 | 1.000 |
|  | 49 | 47 | 14.957 | -1.309 | 0.352 | 0.954 | 1.000 | 1.000 |
|  | 44 | 36 | 13.567 | -0.151 | 0.368 | 0.956 | 1.000 | 1.000 |
|  | 12 | 10 | 12.743 | -1.041 | 0.422 | 0.958 | 1.000 | 1.000 |
|  | 16 | 15 | 13.024 | -2.103 | 0.314 | 0.963 | 1.000 | 1.000 |
|  | 16 | 14 | 14.978 | 0.451 | 0.451 | 0.964 | 1.000 | 1.000 |
|  | 25 | 24 | 14.189 | -1.266 | 0.297 | 0.966 | 1.000 | 1.000 |
|  | 11 | 10 | 12.721 | -2.461 | 0.333 | 0.967 | 1.000 | 1.000 |
|  | 16 | 14 | 11.697 | -2.340 | 0.319 | 0.977 | 1.000 | 1.000 |
|  | 32 | 27 | 14.048 | 1.382 | 0.356 | 0.978 | 1.000 | 1.000 |
|  | 18 | 15 | 12.468 | -0.323 | 0.352 | 0.979 | 1.000 | 1.000 |
|  | 12 | 12 | 14.770 | 0.261 | 0.455 | 0.981 | 1.000 | 1.000 |
|  | 20 | 17 | 11.075 | -1.289 | 0.324 | 0.983 | 1.000 | 1.000 |
|  | 27 | 26 | 13.910 | -1.350 | 0.305 | 0.984 | 1.000 | 1.000 |
|  | 38 | 33 | 13.779 | -1.584 | 0.307 | 0.984 | 1.000 | 1.000 |
|  | 16 | 13 | 14.218 | -1.899 | 0.282 | 0.986 | 1.000 | 1.000 |
|  | 11 | 10 | 11.861 | -2.324 | 0.289 | 0.990 | 1.000 | 1.000 |
|  | 23 | 23 | 10.450 | -1.349 | 0.257 | 0.990 | 1.000 | 1.000 |
|  | 40 | 33 | 14.994 | -2.313 | 0.235 | 0.990 | 1.000 | 1.000 |
|  | 10 | 8 | 14.154 | -1.625 | 0.429 | 0.991 | 1.000 | 1.000 |
|  | 11 | 10 | 6.820 | -2.475 | 0.333 | 0.991 | 1.000 | 1.000 |
|  | 27 | 22 | 13.761 | -0.577 | 0.325 | 0.992 | 1.000 | 1.000 |
|  | 20 | 17 | 9.568 | -1.407 | 0.265 | 0.993 | 1.000 | 1.000 |
|  | 10 | 10 | 12.813 | 1.049 | 0.422 | 0.995 | 1.000 | 1.000 |
|  | 10 | 10 | 14.416 | 0.274 | 0.333 | 0.996 | 1.000 | 1.000 |
|  | 12 | 10 | 9.537 | -0.391 | 0.289 | 1.000 | 1.000 | 1.000 |
|  | 34 | 28 | 6.364 | -1.075 | 0.238 | 1.000 | 1.000 | 1.000 |
|  | 40 | 37 | 14.485 | -1.225 | 0.219 | 1.000 | 1.000 | 1.000 |
| **Mammals** |  |  |  |  |  |  |  |  |
|  | 16 | 14 | 7.816 | -2.188 | 0.789 | 0.035 | 0.664 | 0.664 |
|  | 11 | 10 | 14.915 | 0.086 | 0.867 | 0.128 | 1.000 | 0.668 |
|  | 59 | 49 | 12.696 | -3.705 | 0.522 | 0.151 | 1.000 | 0.668 |
|  | 24 | 21 | 14.744 | -3.205 | 0.632 | 0.151 | 1.000 | 0.668 |
|  | 15 | 15 | 12.592 | -2.461 | 0.708 | 0.176 | 1.000 | 0.668 |
|  | 15 | 14 | 14.491 | -1.629 | 0.750 | 0.247 | 1.000 | 0.781 |
|  | 15 | 12 | 13.153 | -1.578 | 0.697 | 0.360 | 1.000 | 0.957 |
|  | 15 | 12 | 11.381 | -1.391 | 0.626 | 0.507 | 1.000 | 0.957 |
|  | 19 | 16 | 12.824 | -1.016 | 0.582 | 0.538 | 1.000 | 0.957 |
|  | 19 | 18 | 14.899 | -1.065 | 0.599 | 0.538 | 1.000 | 0.957 |
|  | 10 | 8 | 13.779 | -0.331 | 0.617 | 0.587 | 1.000 | 0.957 |
|  | 10 | 8 | 6.687 | 0.954 | 0.694 | 0.604 | 1.000 | 0.957 |
|  | 11 | 9 | 12.766 | -0.723 | 0.551 | 0.686 | 1.000 | 1.000 |
|  | 15 | 14 | 14.514 | 0.024 | 0.552 | 0.778 | 1.000 | 1.000 |
|  | 16 | 13 | 12.267 | -1.683 | 0.512 | 0.907 | 1.000 | 1.000 |
|  | 17 | 15 | 12.566 | -2.341 | 0.333 | 0.907 | 1.000 | 1.000 |
|  | 23 | 23 | 12.857 | -2.633 | 0.337 | 0.949 | 1.000 | 1.000 |
|  | 13 | 12 | 8.328 | 0.723 | 0.275 | 0.986 | 1.000 | 1.000 |
|  | 20 | 19 | 14.787 | 1.186 | 0.106 | 1.000 | 1.000 | 1.000 |
